# Risk factors of digital dermatitis in feedlot cattle

**DOI:** 10.1093/tas/txab075

**Published:** 2021-05-13

**Authors:** Julian A Cortes, Anice Thomas, Steve Hendrick, Eugene Janzen, Ed A Pajor, Karin Orsel

**Affiliations:** 1 Faculty of Veterinary Medicine, Department of Production Animal Health, University of Calgary, Calgary, Canada; 2 Coaldale Veterinary Clinic, Coaldale, Canada

**Keywords:** beef cattle, decreased efficiency, lameness, pen condition

## Abstract

Digital dermatitis (**DD**) has been reported in North American feedlots, although risk factors are not well characterized. Our objectives were to analyze: (1) foot and leg conformation and (2) pen hygiene, as potential variables that predispose feedlot cattle to DD. Production parameters in DD-affected cattle were compared with healthy cattle and with those diagnosed with more commonly known infectious lesion foot rot (**FR**). In total, 2,854 feedlot cattle in 11 pens in 2 feedlots were assessed (bi-weekly pen walks) throughout the feeding cycle. Pen condition was categorized as: “dry,” “mud present but has good bedding,” “more mud than bedding,” and “excessive mud.” Gait scoring was competed and cattle with abnormal gait or evident foot lesions (i.e., DD or FR) were restrained in a cattle chute for a close foot inspection (*n*=280), including scoring of foot angle and claw set and hind and side views of rear feet and legs. Cumulative incidence of DD (present or absent) and FR was 2.5% (71/2,854) and 11.6% (331/2,854), respectively. Foot and leg conformation was not significantly different between left and right sides or between cattle with (*n*=71) and without DD (*n*=209). Lameness was diagnosed in only 22% of cattle with DD. Cattle with DD gained 0.27 kg/d less compared with healthy cattle (mean ± SD: 1.29 ± 0.29 vs. 1.56 ± 0.27, *P*<0.05) and 0.4 kg/d less compared with FR (1.29 ± 0.29 vs. 1.69 ± 0.25). Presence of DD was not significantly different between pens with “dry” and “mud present but has good bedding,” but for pens with “more mud than bedding” or “excessive mud,” the risk of cattle having DD cases increased significantly [odds ratio (**OR**)=8.55, confidence interval (**CI**): 4.0–18.4 and OR=14.1, CI: 5.9–33.8, respectively]. In conclusion, it is important to keep good pen conditions to reduce the risk of DD, which can be managed through proper stocking density and strategic bedding, irrespective of foot and leg conformation.

## INTRODUCTION

Lameness, the clinical sign of several diseases and disorders that affect cattle gait (Greenough, 2007), is the second most treated condition in feedlot cattle after bovine respiratory disease (Davis-Unger et al., 2017), negatively affecting health, production, and welfare. Foot lesions cause 70–90% of lameness in dairy (Solano et al., 2016) and beef cattle (Demmans et al., 2014), with digital dermatitis (**DD**), foot rot (**FR**), and interdigital dermatitis reported as main causes of lame feedlot cattle (Teixeira et al., 2010; Refaai et al., 2013).

In dairy and beef cattle, several factors impact the risk of getting foot lesions: in addition to environmental variables, other factors include management (Bruijnis et al., 2012; Jeyaruban et al., 2012; Endres, 2017), genetics (Uggla, 2008), and foot and leg conformation (Ødegård et al., 2014; Pérez-Cabal and Charfeddine, 2016). In dairy cattle, poor foot and leg conformation increased the risk of getting DD, due to physical characteristics of the foot affecting contact with a wet surface (Chapinal, 2013). Compared with cattle without DD, those with DD had longer toes and lower heel height (Laven, 2007; Olechnowicz and Jaśkowski, 2010), resulting in accumulation of wet slurry on the foot. However, research in this area is lacking for beef cattle.

It has been implied that infectious diseases of cattle feet are hygiene-related. In dairy cattle, for example, there was an association between DD and presence of wet slurry on legs (Read et al., 1998; Wells et al., 1999; Relun et al., 2013). Furthermore, constant exposure to watery surfaces softens and erodes, not only the hoof, but also the skin around it, decreasing the functional barrier and increasing chances of bacterial infiltration (Borderas et al., 2004; Tibbetts et al., 2006; Buch et al., 2011).  Therefore, a wet environment is of critical importance and of particular concern during spring and summer for outdoor-housed cattle. For feedlot cattle, both environmental and animal level risk factors are of concern (Vollmar, 2016), although their contribution to DD emergence in feedlot cattle is unknown. Our hypotheses were that muddy pens increase the risk of getting DD and that animals with DD have a less optimal leg and foot conformation compared with healthy animals without DD.

Therefore, our objective was to assess impacts of foot and leg conformation and pen condition separately as predisposing factors that influence presence of DD in feedlot cattle and determine how DD affects average daily gain (**ADG**). Besides, a survey was done with producers and pen checkers regarding their perception of lameness and claw lesions occurrence throughout the feeding cycle.

## MATERIALS AND METHODS

Two finishing feedlots were recruited in southern Alberta with assistance of Coaldale Veterinary Clinic (**CVC**), Coaldale, AB, Canada. One feedlot only had heifers, and the other only steers; both had outdoor housing pens, dirt surface, and protection against the wind. Inclusion criteria were: history of DD, a cattle chute to facilitate hind foot examination, and computerized production records. Fall- and winter-placed cattle were included; however, purebred and dairy-type cattle were excluded. Both feedlots were under the same management: once cattle received standard arrival processing (tagging, implants, and metaphylaxis), heifers and steers were allocated in six and five pens, respectively, and initially fed a starter diet and ultimately an energy-dense, grain-based diet with water available ad libitum. A total of 2,854 cattle were selected for the study and enrolled in the trial (*n*=1,548 heifers; *n*=1,306 steers). Data collection commenced in November 2018 and concluded in November 2019. All procedures were approved by the Veterinary Sciences Animal Care Committee (AC17-0224) and Research Ethics Board (REB18-0490) of the University of Calgary (Calgary, AB, Canada).

### Pen Walks

**Figure 1.**
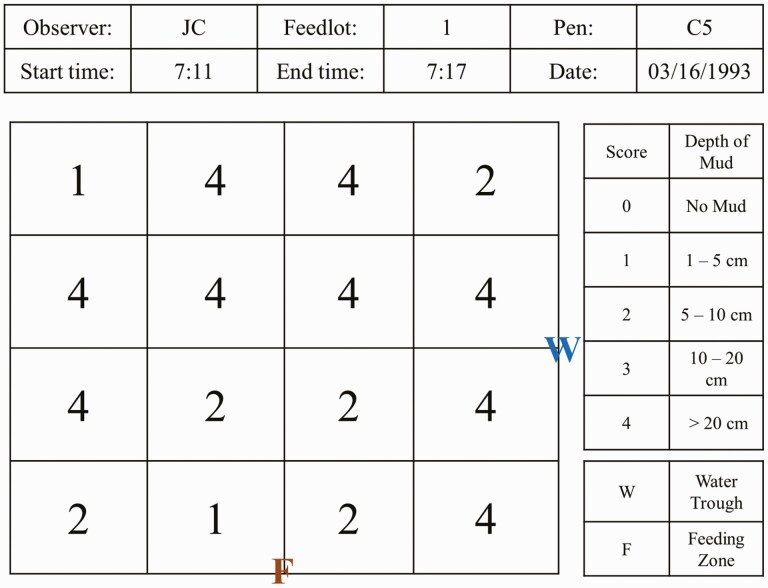
Pen condition scoring.

Graduate students and co-authors Cortes and Thomas (J.A.C. and A.T.) performed a visual inspection of each pen on a bi-weekly basis, from the moment the cattle arrived at the feedlot to the moment they were sent to slaughter. Feet of all animals were inspected within 1 wk of arrival to the feedlot. In each pen, mud depth was measured 16 times at equidistant points:


$$\begin{array}{l} Pen\;condition=\frac{\underset{j}{\sum }X_{j}*f_{j}}{N} \end{array}$$

where *X*_*j*_ are different observations, *f*_*j*_ the relative frequency, and *N* the total number of observations, using the following scale: 0 = no mud; 1 = 1–5 cm; 2 = 6–10 cm; 3 = 11–20 cm; 4 = > 20 cm. An example can be seen in Figure 1.

Every visit to each pen generated a new pen condition score, which was collected from November 2018 to November 2019. Afterwards, the average pen condition score per pen was estimated and was assigned to one of the following groups: <1 = dry pen, which means that, on average, the pen remained dry for the whole period of observation; 1 to 2 = mud present but had good bedding; 2 to 3 = more mud than bedding; and >3 = excessive mud.

During pen walks, a gait score was performed, using the gait scoring system developed by Terrell et al. (2017). Also, leg cleanliness was scored in a subset of the cattle (*n*=361), by inspecting the hind legs from the coronary band to the middle of the tarsal joint, scoring from one to four according to the percentage of the leg covered with manure, dirt, mud: 1= ≤25%, 2= 25–50%, 3=50–75%, and 4=≥75%. This subset of cattle was selected using a simple random sample (every fourth animal) at arrival to the feedlot.

### Foot Inspection

**Figure 2.**
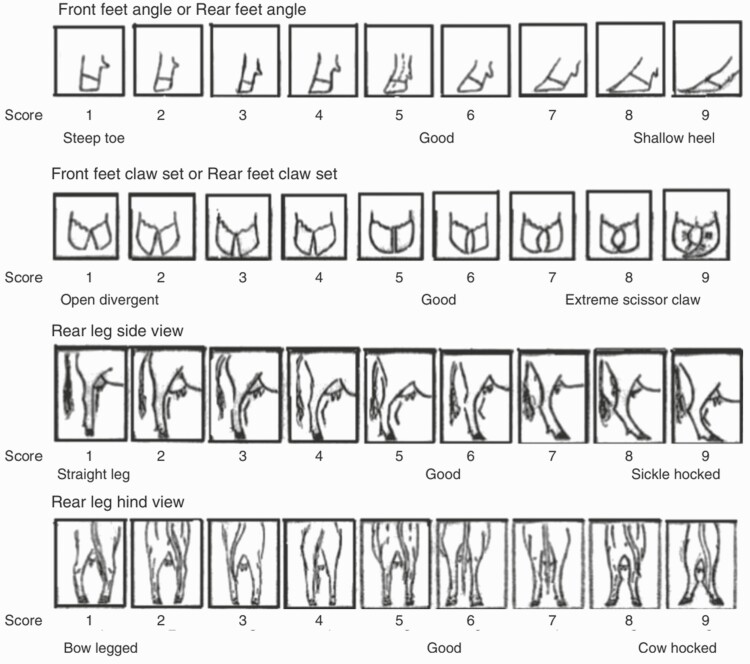
Foot and leg conformation scoring system (Jeyaruban et al., 2012).

A subset of 280 cattle with abnormal gaits and/or a foot lesion was selected during pen walks for a one-time foot inspection in the chute. Physical conformation of hind foot and legs was recorded, as described by Jeyaruban et al. (2012). The four traits evaluated were: rear foot angle (RA), rear foot claw set (RC), rear leg hind view (RH), and rear leg side view (RS). Each of these traits was scored from 1 to 9, with 5 and 6 deemed as ideal scores (Figure 2).

Once cattle were restrained, their rear feet were lifted one at a time and then cleaned using water, a brush, and paper towels to remove manure and dirt. This procedure was performed carefully to avoid causing abrasions. The stage of DD, if present, was scored between heel bulbs using the M-stage system (Döpfer et al., 1997; Berry et al., 2012) to classify lesions, based on the presence of scab-like tissue, proliferation or hyperkeratosis, and/or ulcers. If none was seen, it was deemed as not infected by DD. In addition, the foot was inspected for FR if negative for DD; a diagnosis of FR was based on the presence of both symmetrical swelling and a foul smell (Currin et al., 2009).

### Production Records

Production records were provided by CVC with feedlot workers recording data in a computer database (Athena, CVC). Each animal included in the trial had the following information: sex (steer or heifer), identification, arrival date, arrival weight (**AW**), total days on feed (**DOF**), and date of diagnosis.

### Surveys

Surveys were sent to cattle owners and workers from each feedlot to determine perceptions about lameness. Questions inquired about relative importance of lameness, compared with other common diseases, diagnostic, treatment, and management protocols. Additionally, questions regarding emergence of claw lesions throughout the year were prompted.

### Data Management

Data were extracted from production records, compiled, and organized in a spreadsheet (Microsoft Excel, v.16; Microsoft Corporation, Redmond, WA). The variable terminal weight (**TW**) was calculated by dividing hot carcass weight (**HCW**) by 0.596 (Bartoň et al., 2006). A continuous variable ADG was created [(kg/d); WS – AW/DOF]. Cattle diagnosed for any disease other than DD or FR or cattle that died (*n*=317) were excluded from the analyses.

### Data Analysis

Descriptive statistics were generated for each hind foot and leg conformation, for cattle with and without DD, using STATA 14.1 software (StataCorp LP, College Station, TX). Production parameters were normally distributed using the Shapiro–Wilk test (*P*<0.05); therefore, mean value outputs were generated for ADG, HCW, and TW. All analyses included cattle diagnosed for FR as well.

Foot and leg conformation data were categorized in three groups, where ideal score (5–6) was compared to less ideal scores (1–4: steep toe, open divergent, straight leg, bow legged and 7–9: shallow heel, extreme scissor claw, sickle hocked, cow hocked) for the four traits, to compare between DD and non-DD cattle, using χ ^2^ test, as the assumption of normality was not appropriate (Figure 2).

Furthermore, logistic regression was used to study relationships between DD and pen condition, type of cattle (fall- or winter-placed), and sex. Also, impact on ADG was estimated through linear regression using DD and FR status, pen condition, type of cattle, and sex. For all analyses, *P*<0.05 was considered significant. Clustering by feedlot was not possible as the sex variable also represented feedlot of origin variation (multicollinearity), nor by pens as cattle were frequently resorted in different pens.

For both logistic and linear models, an association between dependent and predictor variables was determined; if the univariate analysis was *P*<0.25, they were included in the model. Backwards elimination was used to assemble the final model. Interactions between terms were evaluated following biological reasoning (i.e., sex and type of cattle) and removed when no significant difference was detected (*P* < 0.05). Pen number was included as a random effect. In addition, a variable was categorized as a confounder if, when removed, a ≥30% change in the estimate of the other predictors was detected.

### Test Accuracy

Besides graduate students J.A.C. and A.T., one technician (LW) with experience in cattle handling was trained to identify pen condition, gait score, foot and leg conformation, and M-stage for DD scoring. Briefly, a kappa statistic was used on a scale from 0 to 1, where a score of 1 is the highest agreement (Viera and Garrett, 2005); agreement of ≥0.6 (substantial agreement) is desirable. The training program included six repeatability sessions (three on-farm and three with videos), which were done every 45 d after the start of the project to sustain agreement. The rate of agreement was calculated using the number of agreements/number of observations * 100.

### Historical Database Analysis

In addition to the field analysis, both feedlots included in the study allowed a historical database analysis for the evaluation of DD seasonality, as it was mentioned by pen-checkers that diagnosis of foot lesions was more common during spring and summer, being consistent with the literature on FR on cattle, sheep, and goats (Rhinehart, 2010; Pezzanite et al., 2014). The dataset was provided by CVC, with feedlot workers recording data in a database (Fusion, CVC). Data were available for a 3-yr interval (2016–2018). All cattle in the dataset had cattle identification, arrival date and AW, DOF, date of slaughter, HCW, and sex recorded. Cattle that were treated also had information regarding diagnosis and date of diagnosis.

A total of 77,115 cattle were part of this historical analysis, of which 894 cattle were recorded as DD-positive. A new variable was created to classify the DD occurrence throughout the feeding cycle in seasons: spring (March 20 to June 20), summer (June 21 to September 21), fall (September 22 to December 20), and winter (December 21 to March 19), and it was further analyzed using the χ ^2^ test. The analysis would reveal whether emergence foot lesions diagnosed during pen walks overlay with season of diagnosis in the historical database.

## RESULTS

### Herd Characteristics

A total of 2,854 feedlot cattle were scored on 2 feedlots (Table 1), with an average of 259 ± 67 cattle per pen. Feet inspection of cattle at arrival showed no feet lesions. Cumulative incidence of DD (present or absent) and FR was 2.5% (71/2,854) and 11.6% (331/2,854) respectively, throughout the feeding cycle, for pens included in the trial (Table 2). The incidence proportion was 24.8 and 116 cases per 1,000 cattle for DD and FR, respectively, or an average of 2.1 per 1,000 cattle per month for DD and 9.7 per 1,000 cattle per month for FR.

**Table 1.** Descriptive statistics of production parameters on both feedlots

Feedlot	*N*	AW^1^ (kg)	95% CI2	DOF^3^ (d)	95% CI	ADG^4^ (kg/d)	95% CI	TW^5^ (kg)	95% CI
1	1,306	348	343–353	217	214–220	1.7	1.69–1.71	714	711–717
2	1,548	313	309–317	226	223–229	1.46	1.45–1.47	640	637–644

1 kg = 2.2 pounds.1AW, arrival weight.2CI, confidence interval. 3DOF, days on feed. 4ADG, average daily gain. 5TW, terminal weight.

**Table 2.** Descriptive statistics for healthy, DD, and FR cattle stratified by sex

Sex	Health status^1^	*n*	AW^2^ (kg)	95% CI	ADG^3^ (kg/d)	95% CI	TW^4^ (kg)	95% CI
Heifers	Healthy	1,366	313	308–317	1.44	1.43–1.46	637	634–641
	DD	51	289	265–313	1.28	1.21–1.36	607	584–629
	FR	129	326	313–339	1.68	1.67–1.69	687	680–695
Steers	Healthy	1,086	350	344–355	1.7	1.69–1.71	715	712–718
	DD	20	383	347–419	1.3	1.14–1.47	634	605–662
	FR	202	335	323–347	1.7	1.69–1.73	717	713–721

1 kg = 2.2 pounds.^1^DD, digital dermatitis; FR, foot rot.

^2^AW, arrival weight.

^3^ADG, average daily gain.

^4^TW, terminal weight.

If >5% of total cattle in a pen was pulled for foot lesions, a footbath (5% CuSO4) was used. Agreement among the three observers was substantial: ≥78% for pen condition (*n*=7 pens); ≥71% for gait score (*n*=29 cows); and ≥63% for DD stages (*n*=42 lesions). For foot and leg conformation, trained personnel (J.A.C. and A.T.) had 77% rate of agreement. Pen size ranged between 6,000 and 8,000 m^2^.

### DD and Foot and Leg Conformation

Cattle diagnosed with FR were excluded from foot and leg conformation analyses, as acute symptoms typical of FR affect assessment. For all four foot and leg conformation traits, there was no significant difference between cattle with and without DD, nor between right and left hind legs; therefore, only measures of the left hind leg were included in the analysis (Boelling and Pollott, 1998). Of the 71 cattle with DD, 16 had a gait score between 1 and 2; and of the 209 cattle without DD, 78 had a gait score of 1 or 2 (mildly and moderately lame).

### DD and Pen Condition

Risk of getting DD (Table 3) was not different between steers and heifers, nor between fall- and winter-placed cattle (*P* < 0.92 and 0.17, respectively). Although there was no significant difference between pen conditions “dry” and “mud present but has good bedding,” for pens with “more mud than bedding,” the risk of having DD cases increased significantly [odds ratio (**OR**)=8.45, confidence interval (**CI**): 3.9–18.5], as well as with “excessive mud” (OR=13.9, CI: 5.7–33.8), relative to “dry.”

**Table 3.** Final logistic regression model for DD with animal and pen-level factors in two feedlots (*N*=2,854)

Risk factor	Odds ratio	SEM	95% CI^1^	*P*-value
Sex				
Heifers	Ref^2^			
Steers	0.96	0.38	0.44–2.08	0.92
Type^3^				
Fall	Ref^2^			
Winter	1.63	0.57	0.81–3.25	0.17
Pen condition^4^				
1	Ref^2^			
2	0.37	0.40	0.05–3.05	0.36
3	8.45	3.37	3.87–18.53	<0.001
4	13.9	6.33	5.67–33.82	<0.001
Pen of origin^5^	0.99	0.06	0.87–1.13	0.87
Baseline odds	0.005	0.002	0.02–0.1	

^1^95% Wald confidence interval.

^2^Reference group used for comparisons.

^3^Type of cattle: fall- or winter-placed calves.

^4^Pen condition: 1=dry, 2=more bedding than mud, 3=more mud than bedding and 4=excessive mud.

^5^Pen of origin as a variable with random effect.

No statistically significant relationship was found between leg cleanliness and lesion presence or gait score.

### DD and Leg Cleanliness

The subset of cattle included in the leg cleanliness analysis did not have enough statistical power to compare between DD and healthy cattle; therefore, these data were excluded from the subsequent analysis.

### DD and ADG

Cattle diagnosed with DD had lower ADG and HCW, relative to healthy (*P* < 0.05) or FR cattle (*P* < 0.05). Besides, cattle with FR had a great ADG (1.69 ± 0.2 kg/d, *P* < 0.05), compared with healthy cattle. No statistically significant difference was found between AW of healthy, DD, and FR animals (Table 4).

**Table 4.** Descriptive statistics of production parameters of feedlot cattle with DD or FR relative to healthy cattle (mean ± SD)

Health status^1^	*N*	AW^2^ (kg)	ADG^3^ (kg/d)	DOF^4^	HCW^5^ (kg)	TW^6^ (kg)
Healthy	2,452	329 ± 92	1.56 ± 0.27^a^	222 ± 57	401 ± 43^a^	672 ± 71^a^
DD	71	315 ± 93	1.29 ± 0.29^b^	232 ± 62	367 ± 45^b^	614 ± 75^b^
FR	331	331 ± 82	1.69 ± 0.25^c^	221 ± 49	421 ± 24^c^	705 ± 40^c^

^a-c^Within a column, values without a common superscript differed (*P* < 0.051). No superscript means no significant difference.1 kg = 2.2 pounds.

^1^DD, digital dermatitis; FR, foot rot.

^2^AW, arrival weight.

^3^ADG, average daily gain.

^4^DOF, days on feed.

^5^HCW, hot carcass weight.

^6^TW, terminal weight.

Cattle with DD had a lower ADG compared with healthy cattle (−0.14 kg/d, P<0.05); whereas those diagnosed with FR had a greater ADG (+0.12 kg/d, *P* < 0.05) relative to healthy or DD-affected cattle (Table 5). Winter-placed cattle had a higher ADG relative to fall-placed cattle, being +0.14 and +0.07 kg/d, respectively, for steers and heifers (*P* < 0.05).

**Table 5.** Linear regression model for ADG with animal and pen-level factors

ADG^1^	Coefficient	SEM	95% CI		*P*-value
Healthy^2^	Ref^3^				
DD	−0.14	0.03	−0.19	−0.09	<0.001
FR	0.12	0.01	0.09	0.14	<0.001
Sex and type^4^					
FPH	Ref^3^				
WPH	0.07	0.01	0.05	0.1	<0.001
FPS	0.24	0.01	0.20	0.27	<0.001
WPS	0.38	0.02	0.34	0.42	<0.001
Pen condition^5^					
1	Ref^3^				
2	−0.03	0.01	−0.05	−0.01	0.5
3	−0.14	0.01	−0.16	−0.12	<0.001
4	−0.38	0.02	−0.42	−0.36	<0.001
Pen of origin^6^	−0.02	0.002	−0.02	−0.01	<0.001
Herd ADG	1.58	0.01	1.55	1.6	<0.001

^1^ADG, average daily gain.

^2^DD, digital dermatitis; FR, foot rot.

^3^Reference group used for comparisons.

^4^FPH, fall-placed heifer; WPH, winter-placed heifer; FPS, fall-placed steer; WPS, winter-placed steer.

^5^Pen condition: 1=dry, 2=more bedding than mud, 3=more mud than bedding, and 4=excessive mud.

^6^Pen of origin as a variable with random effect.

When comparing each of the pen conditions relative to “dry,” there was no significant difference in ADG for pens with “mud present but has good bedding.” However, pens with “more mud than bedding” and “excessive mud” caused a considerable decrease in ADG (−0.14 and −0.38 kg/d, *P* < 0.05, respectively), relative to pens categorized as “dry.” There was no significant interaction between variables included in the final model.

### Historical Data Analysis and Disease Seasonality

**Figure 3.**
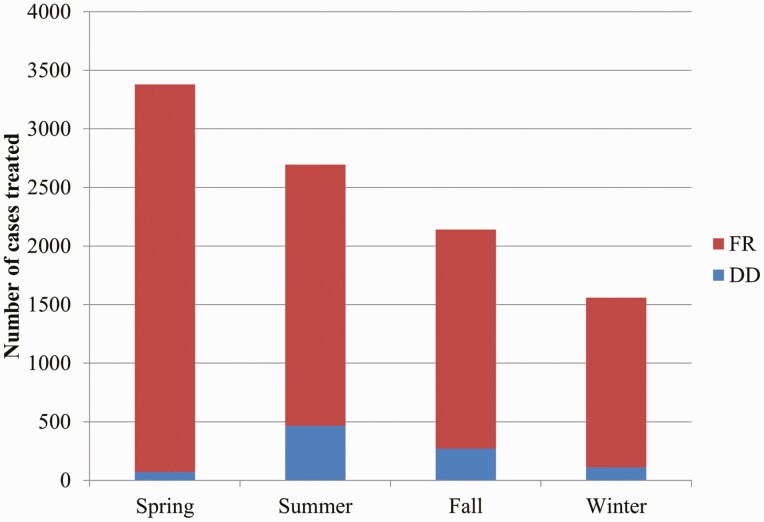
Number of cases treated of DD and FR in each season during a 3-yr interval (2016–2018). DD, digital dermatitis; FR, foot rot.

Cases by season can be seen in Figure 3. There was a statistically significant difference in the number of cases of FR during spring, relative to the rest of the seasons (*P* < 0.05), as well as a statistically significant difference of DD cases during summer, relative to the rest of the seasons (*P* < 0.05).

**Figure 4.**
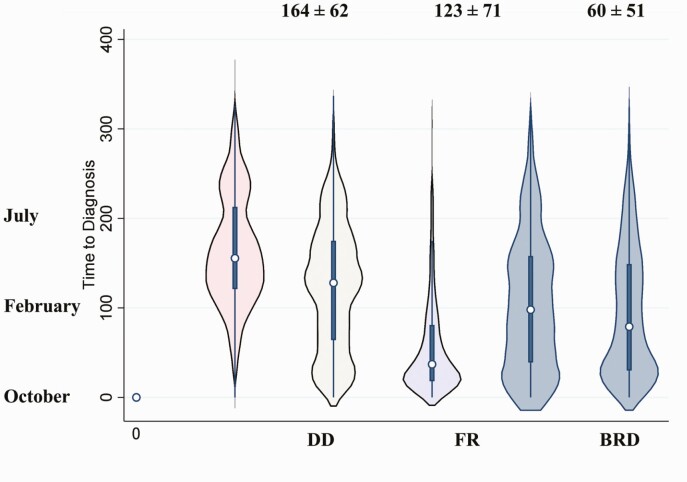
Average DOF at diagnosis of DD, FR, and bovine respiratory disease (BRD) from a 3-yr interval dataset of the two feedlots.

During spring, 4.0% and 37.6% of the DD and FR cases, respectively, were diagnosed, whereas during summer, 49.2% of all DD cases, together with 25.5% of all FR cases, were diagnosed in that 3-yr interval, as shown in Figure 4.

## DISCUSSION

This study followed cattle from two feedlots throughout the feeding cycle, from November 2018 to November 2019, to study type of cattle, sex, foot and leg foot conformation, and pen condition as potential risk factors for getting DD. In addition, association between DD and ADG was evaluated.

Cattle with DD showed no significant difference for foot and leg conformation relative to healthy cattle. In dairy cattle, for example, associations between foot diseases and foot and leg conformation varied among breeds (Uggla, 2008; Stoop et al., 2010; Ødegård et al., 2014). However, given the genetic diversity handled in commercial feedlot cattle, comparison of foot and leg conformation between breeds was not performed.

Hygiene (pen condition) had a significant role in the emergence of DD. The problem worsened as pen condition was poor for a consecutive interval, meaning more mud than bedding and/or excessive mud. Similarly, in dairy cattle, hygiene affected the risk of getting DD (Relun et al., 2013; Solano et al., 2015). Mud not only increased risk of getting DD, but also decreased ADG, consistent with the literature. According to Sweeten et al. (2014), 10–20 cm (4–8 in) of mud increases feed conversion rate by 13%, whereas >30–60 cm (12–24 in) decreases ADG by 25%. However, the study design does not allow to properly quantify how much of the ADG is affected by foot conditions and how much due to the pen condition. In that regard, energy expenditure of cattle likely increases when they walk on a muddy vs. even surface, as the effort to move increases (Dijkman and Lawrence, 1997). Furthermore, a wet and muddy coat likely increases energy requirements for maintenance and therefore increases feed conversion rate (Mader, 2014).

Although there are several ways to reduce excess of moisture in the pen, they can be divided into two main categories: stocking density and pen condition. Regarding density, dry areas should be provided as much as possible, to facilitate dispersion and rest (Mader, 2014). Furthermore, mounds help cattle to stay dry throughout the feeding cycle (Mader and Griffin, 2015), together with more frequent bedding during wet periods.

Unlike DD, cattle that had FR had a higher ADG. The chronic nature of DD may allow it to go unnoticed for longer intervals (Laven and Proven, 2000), thereby causing greater effects on production parameters, relative to acute diseases such as FR, which is easier to identify in the field followed by effective interventions. Furthermore, in this study, only 22% of the cattle with DD were scored as lame, meaning that DD lesions can be present without causing lameness. In contrast, FR is speculated to be responsible for up to 75% of all lameness cases (Currin et al., 2009).

Historical analysis revealed increased DD diagnosis in summer relative to spring, but vice versa for FR. Therefore, we speculated that pen condition affected not only DD emergence but also detection, as wet slurry in contact with the foot decreases accuracy of diagnosis. Besides, lesions development could also play a role in DD detection. In dairy cattle, the average time for a DD lesion to develop was 133 d (Krull et al., 2016), whereas the average time for DD in feedlot cattle is yet to be determined. Therefore, further studies on DD detection at early stages in feedlot cattle are encouraged.

Due to the nature of this study, there were limitations and potential biases. The main one was the number of feedlots assessed, as there is a chance that they are not representative of the entire feedlot industry in North America, hence limiting external validity. Those feedlots enrolled had either heifers or steers (but not both) affected the estimation of the random effect caused by farm in the models used. Furthermore, even when a diagnosis of lesions was done by more than one observer and kappa score calculated, there was always a risk of differential misclassification.

It is noteworthy that this is one of a few longitudinal studies evaluating DD and its potential risk factors in the Canadian feedlot industry, as well as its impact on ADG. With increasing evidence of how pen condition has an economic impact, perhaps foot lesions can be mitigated by management of bedding, particularly during wet seasons. Future research to assess bedding management and its effects on production and cattle health is required.
